# When to start clean intermittent catheterization (CIC) in children with neurogenic bladder dysfunction

**DOI:** 10.1590/S1677-5538.IBJU.2020.0989.1

**Published:** 2021-12-21

**Authors:** Atila Rondon

**Affiliations:** 1 Universidade do Estado do Rio de Janeiro Rio de Janeiro RJ Brasil Universidade do Estado do Rio de Janeiro - Uerj, Rio de Janeiro, RJ, Brasil; 2 Hospital Federal Cardoso Fontes Rio de Janeiro RJ Brasil Serviço de Urologia, Hospital Federal Cardoso Fontes, Rio de Janeiro, RJ, Brasil

## COMMENT

The interest in the management of neurogenic bladder dysfunction in children is increasing due to the development of strategies to protect the upper urinary tract and to avoid or postpone the need for future surgical interventions. New centers are providing this care and this article represents an important document bringing necessary update on this topic, gathering different international guidelines and original studies discussed with a panel of experts. This is a unique opportunity to have the best scientific evidence available.

This paper elegantly shows with extensive scientific evidence how to evaluate the child from the start, suggesting when to perform ultrasonography, urodynamic study, cystourethrography, DMSA scans and when to star clean intermittent catheterization (CIC) (1).

Proactive approach seems to be better than expectant approach, with the early use of clean intermittent catheterization and pharmacotherapy. ESPU Guidelines (2) and ICCS (3) recommendations suggest CIC should be started soon, after birth. This would diminish renal complications and the need for future augmentation. This is also better accepted by parents and patients, among other advantages. The authors scrutinized the indications and exceptions in this matter.

It would be reasonable to assume that the data available would convince most groups to follow this strategy but the best moment to apply these measures has been subject to controversy.

The authors suggest performing a first urodynamic study within 6 weeks after closure of the neural tube defect, looking for a subgroup of patients with greater risk due to reduced bladder capacity and compliance and high detrusor leak point pressure. Urodynamic studies in children are challenging and it is of utmost importance to know what to look for.

The authors went beyond, analyzing the updated scientific evidence on CIC indications, how to do it, how to train parents and which catheters to use in different scenarios, from diameter to hydrophilic coatings. Pharmacological therapies have also been addressed. A long list of studies are presented showing indications and results with the role of anticholinergics, beta3 agonists, alfa-blockers and botulinum toxin.

This is a must-read paper for those dealing with children with neurogenic bladder.
